# An improved method for the detection of double-stranded RNA suitable for quality control of mRNA vaccines

**DOI:** 10.1093/procel/pwae043

**Published:** 2024-08-21

**Authors:** Jingjing Liu, Tao Zheng, Lingjie Xu, Zhicai Chen, Kunkun Zhang, Xiangxi Wang, Xiaoyu Xu, Yuhua Li, Yao Sun, Ling Zhu

**Affiliations:** Department of Arbovirus Vaccine, National Institutes for Food and Drug Control, Beijing 102629, China; CAS Key Laboratory of Infection and Immunity, National Laboratory of Macromolecules, Institute of Biophysics, Chinese Academy of Sciences, Beijing 100101, China; Nanjing Vazyme Biotechnology Co., Ltd., Nanjing 210046, China; Nanjing Vazyme Biotechnology Co., Ltd., Nanjing 210046, China; Nanjing Vazyme Biotechnology Co., Ltd., Nanjing 210046, China; CAS Key Laboratory of Infection and Immunity, National Laboratory of Macromolecules, Institute of Biophysics, Chinese Academy of Sciences, Beijing 100101, China; Nanjing Vazyme Biotechnology Co., Ltd., Nanjing 210046, China; Department of Arbovirus Vaccine, National Institutes for Food and Drug Control, Beijing 102629, China; CAS Key Laboratory of Infection and Immunity, National Laboratory of Macromolecules, Institute of Biophysics, Chinese Academy of Sciences, Beijing 100101, China; CAS Key Laboratory of Infection and Immunity, National Laboratory of Macromolecules, Institute of Biophysics, Chinese Academy of Sciences, Beijing 100101, China

In more than 200 years since Edward Jenner successfully developed the first vaccine for combating smallpox, vaccines have played pivotal roles in preventing outbreaks of several infectious diseases, and it could even be asserted that each one of us has benefited from them. The recent severe acute respiratory syndrome coronavirus 2 (SARS-CoV-2) outbreak has also demonstrated the importance of vaccines in interrupting the transmission of infectious diseases (Deng and Peng, 2020). Among the different technology routes explored for preparing SARS-CoV-2 vaccines, mRNA vaccines are emerging as a powerful weapon in the fight against SARS-CoV-2 (Sumirtanurdin and Barliana, 2021). The licensing and application of SARS-CoV-2 vaccines by Moderna and Pfizer have fully demonstrated the applicability and technological advancement of the mRNA vaccines (Xia, 2021). Considering the significant contributions of mRNA vaccines in combating the coronavirus disease 2019 (COVID-19) pandemic, the 2023 Nobel Prize in Physiology or Medicine was awarded for the discoveries concerning nucleoside base modifications that enabled the development of effective mRNA vaccines against COVID-19. When compared to traditional vaccines the clinical risks of mRNA vaccines have not been sufficiently investigated. During the *in vitro* transcription (IVT) step, the conversion of DNA templates into mRNA is performed by phage RNA polymerases like T7 RNA polymerase (T7 RNAP), which is renowned for its high fidelity in RNA synthesis. However, the biological process is not entirely error-proof. It can sometimes lead to the generation of certain mRNA byproducts; in particular, the formation of double-stranded RNA (dsRNA), which is inherently immunogenic, triggering an inflammatory response in the body. Such a side-effect is not desirable in mRNA therapeutic applications (Mu and Hur, 2021). The impact of dsRNA on mRNA therapy efficacy remains a subject that has not been comprehensively explored. One of the major hindrances to such investigations is the inadequate sensitivity and accuracy of current dsRNA detection methods.

dsRNA is a byproduct of the *in vitro* transcription performed during the preparation of mRNA for the vaccine formulation. When the mRNA component of the vaccine enters inside the cell, dsRNA enters the cell with it. This dsRNA causes an intracellular inflammatory response mainly through two signaling pathways, the TLR3 as well as the RIG-I/MDA5 pathway, which ultimately activate the expression of interferons and other proinflammatory cytokines (Kato et al., 2011; Pohar et al., 2013). At the individual level, this manifests as a side-effect of vaccination, with localized redness and swelling, increased body temperature, and persistent pain. In some cases, it can be life-threatening. There are several mechanisms of dsRNA generation in the IVT process of mRNA vaccine preparation. Abortive fragments generated early in transcription may mediate transcriptional elongation in the opposite direction by the T7 RNAP, resulting in the formation of shorter dsRNA fragments. Conversely, if transcription is not stopped in time at the end point, then mRNA tails fold back and the T7 RNAP continues to use the mRNA as a template for transcription to generate long fragments of dsRNA (Dousis et al., 2023). These two types of dsRNAs characterized by significant differences in lengths, should be of particular concern. Given the potential clinical risks associated with dsRNA, it is imperative to implement robust measures for the detection and control of dsRNA during the production and preparation of mRNA vaccines. However, the existing methodologies for dsRNA detection have many limitations. For instance, Pfizer’s approved mRNA vaccine relies on the Dot-Blot method for dsRNA detection, a technique characterized by its crudeness and limited capacity for only semi-quantitative detection. This approach, perhaps necessitated by the urgency of the epidemic at that time, is unsuitable for meeting the rigorous demands of mRNA vaccine release and clinical research. Consequently, there is an urgent need within the realm of mRNA research and application for the development of an accurate and sensitive dsRNA detection method.

In this study, considering the relatively weak immunogenicity of dsRNA itself, we conducted multiple rounds of systematic antibody screening to ultimately obtain optimal antibodies targeting dsRNA. Subsequently, we employed Cryo-electron microscopy (Cryo-EM) techniques and protein–nucleic acid complex prediction models to analyze the structure of the dsRNA–antibody complexes. At the molecular level, we delineated the basis for our antibody pair binding to dsRNA. Following this, we prepared and screened ideal high-stability dsRNA detection standards which ensured their availability for the establishment of a detection method and validation. Subsequently, for the quantitative detection, we established a systematic detection method based on the enzyme-linked immunosorbent assay (ELISA) technique which is generally more sensitive and can be adopted to a high-throughput mode. Finally, we demonstrated the accuracy and precision of our dsRNA detection method through a comprehensive methodological validation. Our study contributes scientific value to the study of dsRNA and the subsequent investigation of mRNA vaccines.

In order to establish a better dsRNA detection method, we first carried out the synthesis of dsRNA antigen. IVT was chosen to synthesize the positive and negative strands respectively, which were then annealed to form dsRNA, and finally digested by single-stranded RNAase and DNase I to remove the single-stranded RNA (ssRNA) or the template to obtain dsRNA antigen or the standards (Fig. 1A). The purity of the synthesized dsRNA was more than 95% (Fig. S1A). We decided to establish an ELISA method for the quantitative detection of dsRNA. The most important consideration for such a method was the identification of suitable antibodies that could bind dsRNA. Such antibodies, e.g., J2 and K2, have been reported previously (Bonin et al., 2000; Schonborn et al., 1991; Zangger et al., 2013). We embarked on screening for better dsRNA binding antibodies. The above synthesized dsRNA was immunized in mice, and after three rounds of booster, cell fusion was performed to screen hybridoma cells (Fig. S1B). A total of 119 dsRNA binding antibodies were obtained from 6 mice using the hybridoma technique, including 43 IgG2a, 18 IgG2b, 3 IgG3, 52 IgM, and 3 IgA, and the binding-positive percentage of each subtype of antibody was basically the same. Afterwards, 62 binding-positive antibodies were obtained by ELISA screening assay of which 48 strains were preferably continued for subsequent screening (Fig. 1B). Utilizing the indirect ELISA method, the preferred antibodies were ranked and analyzed based on the EC_50_ values as indicators. Some affinity analysis results are presented in Figs. 1C and S1C. Meanwhile, the 62 binding-positive antibodies were subjected to ELISA assays against ssRNA, double-stranded DNA (dsDNA), and single-stranded DNA (ssDNA) individually, measuring the OD_450_ values. Antibodies were selected based on the criteria that the ratio of OD_450_ > 5 for dsRNA to those for ssRNA (Fig. 1D), dsDNA (Fig. 1E), or ssDNA (Fig. 1F), aiming to obtain antibodies with superior sensitivity and specificity. Combining the aforementioned results, a total of 6 antibodies, namely M2, M5, M37, M52, M58, and M61, were ultimately identified with favorable affinity and specificity. According to the results of epitope competition assays, the six antibodies were categorized into two groups (Table S1). Totally nine possible antibody pairs were assessed using the sandwich ELISA method to measure the response values against dsRNA and ssRNA, respectively. The optimal antibody pair, identified as M2 and M5, was consequently selected (Fig. 1G). The complete screening process is summarized in the funnel-shaped flowchart (Fig. S1D). To further determine the affinity between the antibodies and dsRNA precisely, bio-layer interferometry experiments were conducted. The results indicated that the affinities of both M2 and M5 antibodies for the 40 bp dsRNA are at the nanomolar level (Fig. S1E and S1F).

**Figure 1.**
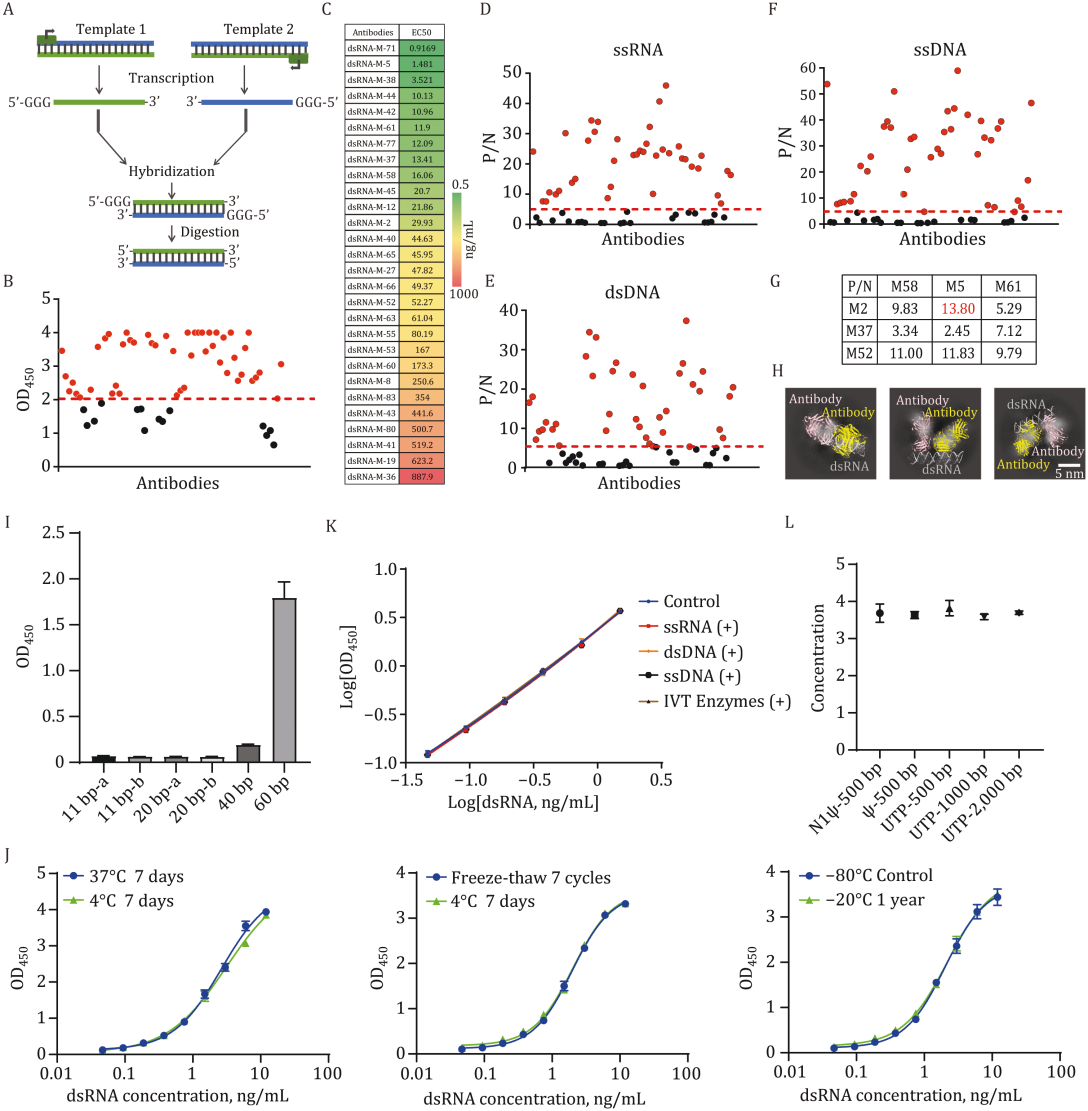
Establishment and methodological validation of the dsRNA detection method. (A) Schematic diagram of the synthesis of dsRNA standards. (B) The indirect ELISA was performed with OD_450_ > 0.2 (2–3 times of negative wells) as the criterion for defining binding-positive antibodies and OD_450_ > 2 as the preferred. A total of 62 (48 preferred) binding-positive antibodies were obtained from 6 immunized mice. (C) Utilizing the indirect ELISA method, the analysis of preferred antibodies is ranked based on EC_50_ values as indicators. (D–F) The ELISA method was employed to determine the reactivity of antibodies with ssRNA (D), dsDNA (E), and ssDNA (F) individually. (G) Nine pairs of antibodies were individually tested for their reactivity with both dsRNA and ssRNA, and the resulting ratios are listed in the table within the Fig. 1G. (H) The fit results of antibody-dsRNA complex into the 2D-classified density maps. The structures of antibodies are predicted with alphafold2 and docked to dsRNA with NPDock. (I) The selectivity of the M2 and M5 antibody pair towards dsRNA with lengths from 11 bp to 60 bp was tested by ELISA. Distinct sequences with the same length are differentiated by labels “a” and “b”. (J) The results of the thermal stability and repeated freeze-thaw stability tests for the samples of dsRNA standards. (K) The impacts of potential interferents such as ssRNA, dsDNA, ssDNA, and IVT enzymes on the dsRNA detection system within the linear range of 0.046–1.5 ng/mL. (L) The sandwich ELISA technique was employed to assess the selectivity of the M2 and M5 antibody pair for dsRNA modified with UTP, ψ, N1-ψ as well as dsRNA of varying lengths.

As for the advantage of M2 and M5 antibody pair in detecting dsRNA, we reasoned that it might be related to the mode of binding of the antibodies to dsRNA. So we tried to analyze the complex structure of antibody-dsRNA by Cryo-EM. Firstly, we incubated dsRNA with purified M2 and M5 Fab fragments as well as dsRNA with single M2 or M5 Fab, followed by Cryo-EM sample preparations and data collection. Analysis of the 2D classifications (Figs. S2 and S3) revealed a clear form of double antibody bound when both M2 and M5 antibodies were present simultaneously. In contrast, this characteristic was not observed when only one type of antibody (M2 or M5) was present alone. Therefore, we believe that the observed complex corresponds to the hetero form. Consistent with expectations, due to the structural flexibility of dsRNA, we did not obtain the high-resolution density map of the complex. As an alternative, we predict the dsRNA-antibody complex structure using a software named NPDock (Tuszynska et al., 2015), which could mimic the interaction between nucleic acid and protein. By analyzing the top 100 best scored predicted binding models, we could deduce that the antibodies do not have a preference for the sequence of the dsRNA, but they have a preference for the structure of the nucleic acid, which is consistent with the previous study. The M5 antibody specifically binds to the minor groove of dsRNA perpendicularly or at an angle of 45° with the CDR region of the heavy chain mediating the main interaction. By contrast, the M2 antibody only binds to the dsRNA at a vertical angle, whose CDR region of the heavy chain binds to the minor groove and the CDR region of the light chain binds to the major groove of the dsRNA (Fig. S4). The predicted structure can fit well with the calculated density map (Fig. 1H). In summary, we could construct a plausible mode of binding of the antibody pair, M2 and M5, with dsRNA through a combination of preliminary Cryo-EM data and modeling studies.

In view of the fact that dsRNAs in organisms are generally above 40 bp to be functionally active as reported previously (Blango and Bass, 2016; Lybecker et al., 2014), we first tested the shortest recognized length for dsRNA using the M2 and M5 antibody pair. From the results, we could see that similar to the classical J2 and K2 antibody pair, our M2 and M5 antibody pair could only recognize dsRNAs larger than 40 bp (Figs. 1I and S5A). This was also consistent with the shortest length required for dsRNA to provoke intrinsic immunity *in vivo*. Next, in order to screen and obtain suitable dsRNA standards, we examined the reactivity of the M2 and M5 antibody pair against dsRNA of various lengths. The results indicated that the M2 and M5 antibody pair exhibited consistent reactivity among dsRNA fragments ranging from 200 bp to 2,000 bp. Therefore, we chose a medium-sized dsRNA of 500 bp as the detection standard for subsequent studies (Fig. S5B). Next, we tested the purity of the synthesized dsRNA standards. The purity was verified to be above 95% both by the agarose gel electrophoresis and the HPLC-SEC, respectively (Fig. S5C and S5D).

Following the confirming of dsRNA standards, we performed a comprehensive methodological validation of the new dsRNA detection assay. First, we assessed the stability of the dsRNA standards. The 37°C acceleration and freeze-thaw stability of the standards were examined by detecting the EC_50_ value of J2 antibody binding to dsRNA at different time points. The results showed that the deviation of EC_50_ of the dsRNA standard was less than 30% after 7 days at 37°C or 7 times of freeze-thawing, indicating that the standard for dsRNA was stable (Fig. 1J). Secondly, in terms of specificity, we verified that the ssRNA, dsDNA, ssDNA, and the *in vitro* transcriptional enzymes used during mRNA preparation did not interfere with the detection system within the linear range of 0.046–1.5 ng/mL (Fig. 1K). As a comparison, we also conducted sensitivity and specificity analyses using the classic J2 antibody with the Dot-Blot method. The results showed that the sensitivity of Dot-Blot was comparatively weak, approximately 6.25 ng/mL (Fig. S6A). Additionally, its detection is susceptible to interference from components such as ssRNA, dsDNA, and ssDNA (Fig. S6B). Thirdly, we employed sandwich ELISA to assess the reactivity of the M2 and M5 antibody pair towards dsRNA modified with UTP, ψ, N1-ψ, as well as dsRNA with different sequences. The results indicated that the M2 and M5 antibody pair exhibited minimal selectivity towards both sequences and base modifications (Fig. 1L). Additionally, we conducted experiments and analyses on the inter-batch and intra-batch precision, accuracy, and total error of the dsRNA quantitative detection method. The results revealed that precision errors were <15% and accuracy errors were <20%, meeting the requirements for application (Table S2). Finally, to assess potential matrix effects, we subjected three distinct mRNA samples to gradient dilutions, determining their concentrations separately and recalculating the original concentrations. Precision errors for the different concentrations were consistently <15%, indicating favorable dilution parallelism for the detection method and minimal impact from matrix effects (Table S3). Despite the aforementioned advantages, it is undeniable that the ELISA assay also has some limitations. In brief, ELISA method is relatively high-cost and time-consuming compared to other dsRNA detection methods such as Dot-Blot. Moreover, the complexity of its components may increase the stability risks. Taking a comprehensive view, the ELISA method we have developed for quantifying dsRNA shows significant improvement over existing quantification methods, thereby advancing the quality control methods for mRNA vaccines. To further elucidate the prospective application of quality control in authentic mRNA vaccines, we quantified dsRNA using our assay on mRNA vaccine products from nine batches across three companies. The results demonstrate high method stability and quantitative consistency with expected values, indicating the suitability of our method for practical vaccine product quality control (Fig. S7).

The detection of dsRNA which is often considered a byproduct of *in vitro* transcription plays a pivotal role in the quality control of mRNA vaccines. This significance stems from its utility in assessing the purity and integrity of the mRNA used in vaccine formulations. Here, we have obtained an optimal antibody pair of dsRNA, M2, and M5, that can be used effectively for the detection of dsRNA. The method developed for the detection of dsRNA exhibits high specificity towards dsRNA, demonstrating robust resistance to interference from matrix components, IVT enzymes, and other potential sources of interference present in the reaction system. Additionally, the method demonstrates high accuracy and precision, making it a promising candidate for future quality control applications in the production of mRNA vaccines. Meanwhile, considering the high affinity and specificity of M2 and M5 antibodies towards dsRNA, beyond their utility in dsRNA detection, it is also expected to be applied to the purification stage to reduce dsRNA contamination in mRNA vaccines.

## Supplementary data

Supplementary data is available at https://doi.org/10.1093/procel/pwae043.

pwae043_suppl_Supplementary_Figures_S1-S7_Tables_S1-S3
