# Supplementary material for: An improved method for the detection of double-stranded RNA suitable for quality control of mRNA vaccines
Source: Protein Cell. 2024 Aug 21;15(11):791–5. doi: 10.1093/procel/pwae043 (PMC11528662; doi:10.1093/procel/pwae043)
Supplement: pwae043_suppl_Supplementary_Figures_S1-S7_Tables_S1-S3 [file pwae043_suppl_supplementary_figures_s1-s7_tables_s1-s3.pdf]

An improved method for the detection of double-stranded RNA suitable for quality control of mRNA vaccines

Jingjing Liu[1]*, Tao Zheng[2]*, Lingjie Xu[3]*, Zhicai Chen[3], Kunkun Zhang[3], Xiangxi Wang[2], Xiaoyu Xu[3#], Yuhua Li[1#], Yao Sun[2#], Ling Zhu[2#]

This file includes:

Materials and methods

Figure S1-S7 and legends

Table S1-S3

Materials and Methods

dsRNA synthesis

Single-stranded RNA was synthesised using *in vitro* transcription, and then denatured by warming to 85°C for 10 min followed by natural annealing to form dsRNA. The *in vitro* transcription reaction kit was purchased from Vazyme Corporation (Cat: DD4201/ DD4202).

Detection of dsRNA by HPLC

The dsRNA samples were diluted into purified water at a final concentration of 100 ng. The assay was performed using a Thermo Vanquish Core instrument with a mobile phase of a concentration of 150 mM phosphate buffer (pH 7.4) and 400 mM NaCl. The column was Sepax SRT SEC-1000 (5 µm, 1000 A, 7.8*300 mm) and the column temperature was 25°C. The flow rate was 0.8 mL/min with the collection wavelength of 260 nm.

Antibody immunization

Antibody immunization was carried out by subcutaneous injection of 100 µg dsRNA for the primary immunization in DBA/2 and BALB/c mice. Subsequently, three booster injections of 50 µg dsRNA were administered intraperitoneally before performing cell fusion. Hybridoma cells were screened, and the antibody supernatant was obtained.

ELISA

The ELISA experiments were conducted as follow. Initially, specific antibodies were diluted to 4 µg/mL in coating buffer and added to a 96-well plate at 0.1 mL/well, incubating at 4°C overnight. After removing the coating solution, wells were washed once with wash buffer (containing 0.05% Tween-20) and then blocked with 0.1 mL blocking solution (1% Casein) at 4°C overnight. Subsequently, 0.1 mL of the antigen-containing test samples were added to each well. Following 1 hour incubation at

37°C, the liquid was discarded, and the wells were washed four times with wash buffer (containing 0.05% Tween-20). Next, 0.1 mL of detection antibody was added and incubated for 1 hour at 37°C. After discarding the liquid, wells were washed four times with wash buffer (containing 0.05% Tween-20) again. Then 0.1 mL of second antibody against detection antibody was added with the same incubation and wash procedure, followed by the addition of 0.1 mL TMB solution to each well and a 15-minute incubation at 37°C. Finally, 0.05 mL of 2 M sulfuric acid was added to each well to terminate the reaction, and the OD_{450nm} values were determined using an enzyme-linked immunosorbent assay reader.

Bio-Layer Interferometry

We employed the Octet R8 instrument (Sartorius) to determine the affinity between dsRNA and Fab fragments. The reaction was conducted in a 96-well plate under the buffer of PBS containing 0.01% Tween-20, with shaking conditions set at 1000 rpm, and the ambient temperature maintained at 26°C. His-tagged Fab fragments (100 μg/mL) were immobilized on the sensor for 300 seconds, followed by equilibration for 600 seconds in PBST Buffer. Subsequently, dsRNA at various concentrations (5.5 nM, 16.5 nM, 49.4 nM, 148.1 nM, 444.4 nM, 1.33 μM, 4 μM) was introduced, allowed to bind for 150 seconds, and then dissociated for 30 seconds. Data were collected and analyzed using the built-in Octet software.

Cryo-EM sample preparation, data collection and data processing

dsRNA sample was incubated with two types of antibodies at 4˚C for 4 h at a ratio of one M2 Fab and one M5 Fab per dsRNA, the final concentration of dsRNA was 2 mg/mL. Next, 3 μL of the sample were applied to holey carbon grids (standard 20 nm Carbon, 1.2 micron Hole Diameter, 1.3 micron Hole Spacing, 300 mesh Gold Grid) which had been

glow-discharged in argon for 20 seconds. The grids were flash cooled in liquid ethane using an FEI Vitrobot Mark IV with a blotting time of 6.5 s at 100% humidity, 4˚C. The data collection was performed using a Titan Krios G3i (FEI) microscope operating at 300 KV with beam image-shift data collection method (Wu, Huang et al. 2019). The datasets were collected under a defocus of -1 μm to -1.5 μm with a pixel size of 0.808 Å. Each movie stack was dose fractionated into 32 movie frames with a total dose of 40 e^{-} $Å^{-2}$. Beam-induced motion correction of all movie stacks was performed with MotionCor2 (Zheng, Palovcak et al. 2017). Aligned micrographs without dose weighting were used to determine the parameters of the contrast transfer function by the program CTFFIND4 (Rohou and Grigorieff 2015). All subsequent steps were performed with cryoSPARC (Punjani, Rubinstein et al. 2017). 2,867,235 particles were picked in 4,512 Micrographs. After 50 rounds of 2D classification, 26 classes of images with clear signals were retained, and the rest were abandoned. The retained particles were subjected to 3D classification.

Dot-Blot

10 μL dsRNA samples were added onto the polyvinylidene difluoride (PVDF) membrane, which was activated by 100% methanol. After loading, the PVDF membrane was blocked in TBS-T buffer containing 5% (w/v) skim milk powder. The membrane was incubated with anti-dsRNA monoclonal antibody J2 (Jena bioscience) for 1 h, then washed with 10 mL TBS-T buffer for 10 min. After washing four times, the membrane was incubated with HRP-conjugated goat anti-mouse IgG (Vazyme) for 1 h. After washing four times, the signal was detected by adding chemiluminescent reagents (Vazyme) and imaged on the ChemiDoc MP Imaging System (Bio-Rad).

Data analysis

All data were analysed using GraphPad Prism 8 and Biostats 1.0 software. Statistical differences were analysed for significance using the unpaired t-test, and errors were analysed using the mean ± SD. $p \leq 0.05$ was considered as significant difference.

Supplemental Reference

Punjani, A., J. L. Rubinstein, D. J. Fleet and M. A. Brubaker (2017). "cryoSPARC: algorithms for rapid unsupervised cryo-EM structure determination." Nature Methods **14**(3): 290-296.

Rohou, A. and N. Grigorieff (2015). "CTFFIND4: Fast and accurate defocus estimation from electron micrographs." J Struct Biol **192**(2): 216-221.

Wu, C., X. Huang, J. Cheng, D. Zhu and X. Zhang (2019). "High-quality, high-throughput cryo-electron microscopy data collection via beam tilt and astigmatism-free beam-image shift." J Struct Biol **208**(3): 107396.

Zheng, S. Q., E. Palovcak, J. P. Armache, K. A. Verba, Y. Cheng and D. A. Agard (2017). "MotionCor2: anisotropic correction of beam-induced motion for improved cryo-electron microscopy." Nat Methods **14**(4): 331-332.

Supplemental Figures

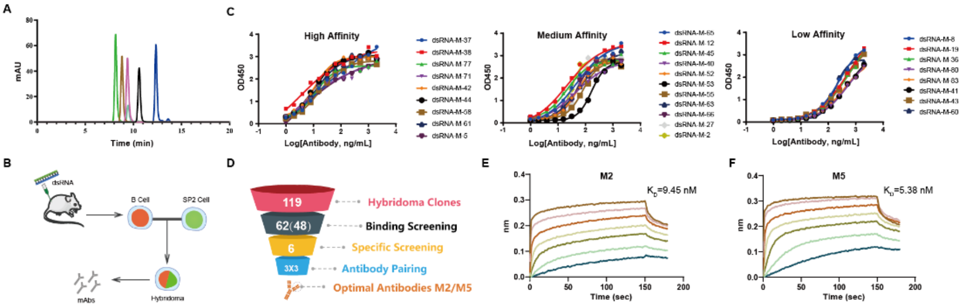

Figure S1. Selection and characterization of dsRNA detection antibodies. (A) HPLC was performed for purity assessment of dsRNA antigen. Cyan represents the target dsRNA antigen, while the rest are dsRNA markers (blue for 100 bp, black for 300 bp, magenta for 500 bp, brown for 700 bp and green for 1000 bp). (B) Model diagram illustrating the antibody generation pattern in mice immunized with synthetically dsRNA. (C) Affinity analysis of the preferred antibodies for dsRNA using the indirect ELISA method. (D) Funnel-shaped flowchart illustrating the selection process for the optimal dsRNA detection antibody pair M2 and M5. (E, F) Affinity analysis of the binding of 40 bp dsRNA to M2 Fab (E) and M5 Fab (F) by BLI. The concentrations from top to bottom are 4 µM, 1.33 µM, 444.4 nM, 148.1 nM, 49.4 nM, 16.5 nM and 5.5 nM.

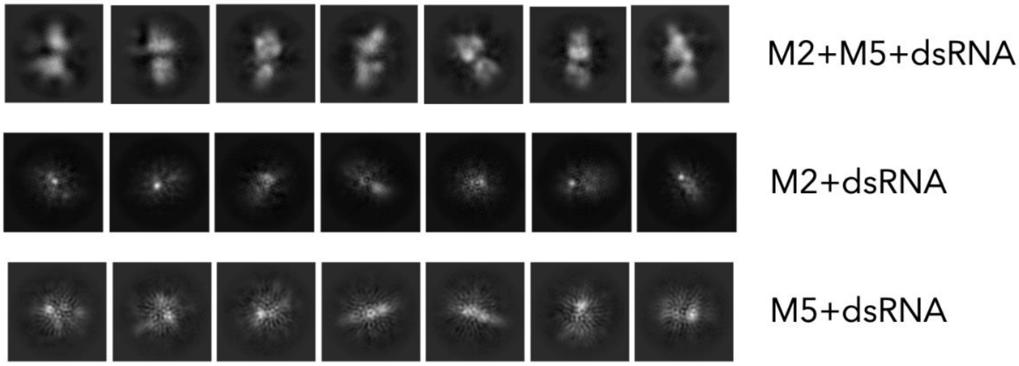

Figure S2. 2D Classifications of anbibody-dsRNA complexes.

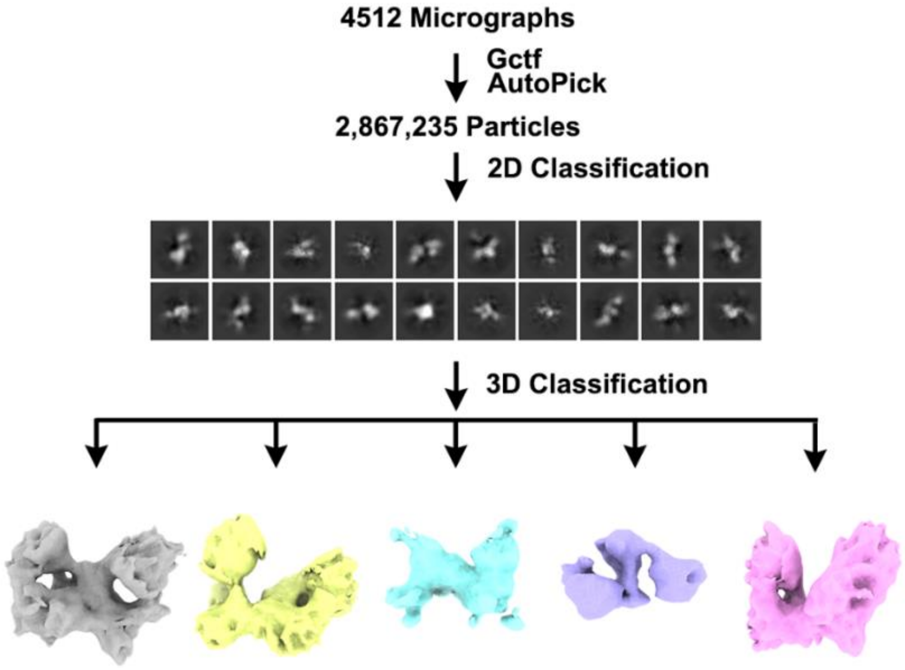

Figure S3. Flowchart of Cryo-EM reconstruction of M2 and M5 antibody pair complex with dsRNA.

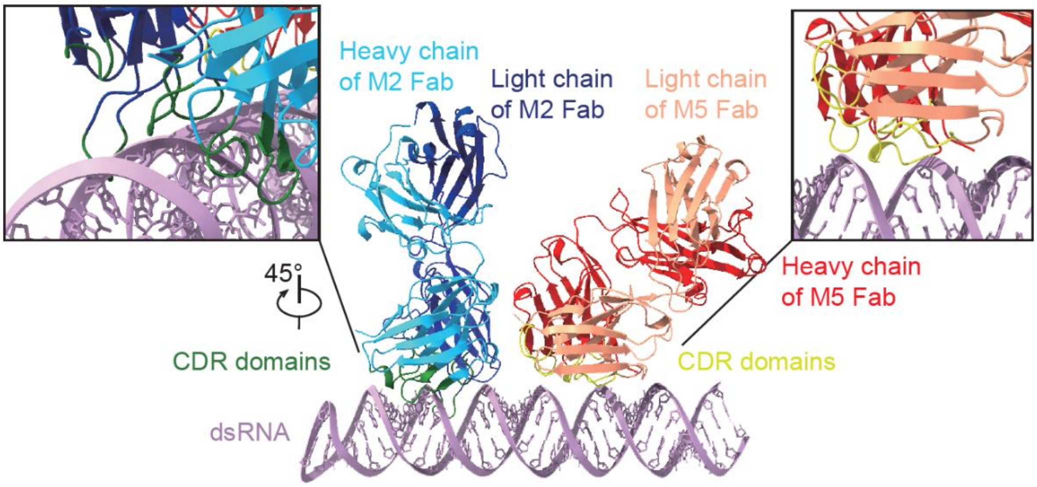

Figure S4. Model for the complex of dsRNA with M2 and M5 antibody pair predicted by NPDock

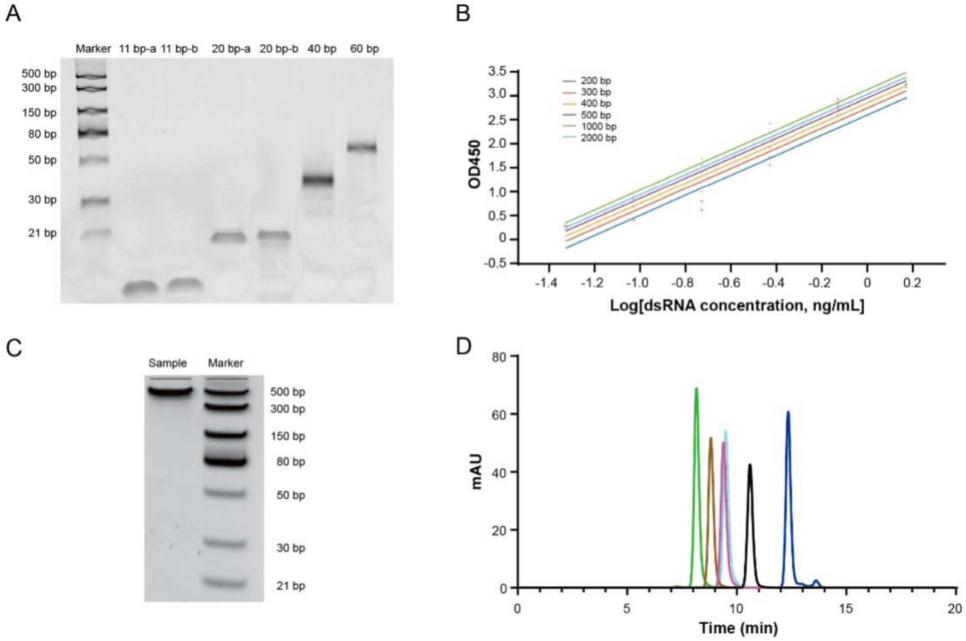

Figure S5. Selection and characterisation of dsRNA standards. (A) Validation of the size and purity of dsRNAs with different lengths by agarose gel electrophoresis. (B) Parallelism experiment to validate the consistent reactivity of M2 and M5 antibody pair with dsRNAs of varying lengths. (C) Validation of the size and purity of dsRNA standards by agarose gel electrophoresis. (D) Validation of the size and purity of dsRNA standards by HPLC. Cyan represents the dsRNA standards, while the rest are dsRNA markers (blue for 100 bp, black for 300 bp, magenta for 500 bp, brown for 700 bp and green for 1000 bp).

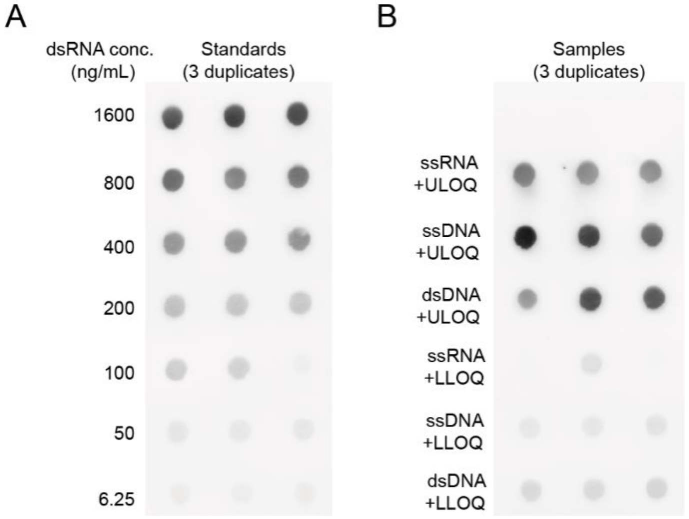

Figure S6. The sensitivity and specificity of Dot-Blot assay. (ULOQ: upper limit of quantification; LLOQ: lower limit of quantification)

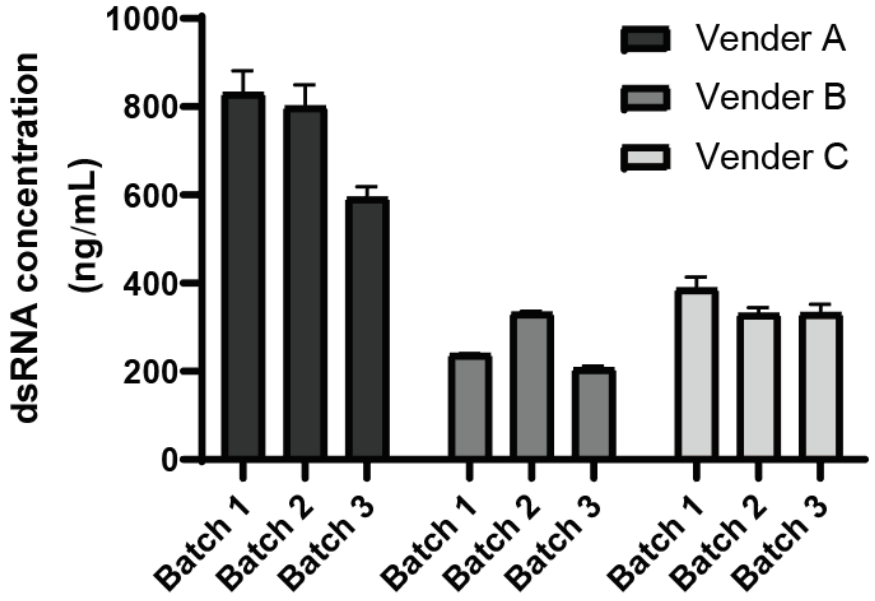

Figure S7. dsRNA detection assay was performed on 9 batches of real vaccine samples from 3 different vaccine manufacturers.

Table S1. Epitope analysis of candidate antibodies

	K1	K2	J2		K2+IgM-HRP	J2-HRP
M-37-HRP	66%	53%	86%	dsRNA-M-37	-11%	87%
M-38-HRP	65%	47%	86%	dsRNA-M-38	8%	94%
M-86-HRP	66%	70%	84%	dsRNA-M-86	-24%	24%
M-89-HRP	54%	35%	84%	dsRNA-M-89	-33%	92%
M-55-HRP	54%	67%	82%	dsRNA-M-55	-27%	70%
M-88-HRP	57%	2%	81%	dsRNA-M-88	50%	93%
M-2-HRP	52%	2%	78%	dsRNA-M-2	-8%	90%
M-67-HRP	67%	59%	78%	dsRNA-M-67	-21%	36%
M-41-HRP	63%	28%	77%	dsRNA-M-41	-31%	84%
M-92-HRP	68%	61%	77%	dsRNA-M-92	-17%	64%
M-57-HRP	64%	61%	76%	dsRNA-M-57	14%	94%
M-82-HRP	15%	9%	38%	dsRNA-M-82	64%	95%
M-66-HRP	2%	60%	60%	dsRNA-M-66	61%	94%
M-59-HRP	44%	19%	30%	dsRNA-M-59	-21%	93%
M-5-HRP	31%	54%	4%	dsRNA-M-5	54%	93%
M-32-HRP	40%	-16%	40%	dsRNA-M-32	-12%	92%
M-31-HRP	42%	0%	48%	dsRNA-M-31	-10%	92%
M-52-HRP	62%	37%	71%	dsRNA-M-52	-18%	92%
M-53-HRP	39%	14%	-1%	dsRNA-M-53	-24%	92%
M-39-HRP	29%	32%	62%	dsRNA-M-39	-35%	90%
M-44-HRP	40%	8%	19%	dsRNA-M-44	-15%	90%
M-87-HRP	53%	-12%	37%	dsRNA-M-87	-23%	90%
M-54-HRP	19%	68%	52%	dsRNA-M-54	-32%	89%
M-58-HRP	35%	6%	-5%	dsRNA-M-58	12%	88%
M-65-HRP	47%	6%	66%	dsRNA-M-65	98%	86%
M-61-HRP	4%	54%	53%	dsRNA-M-61	-19%	84%
M-77-HRP	29%	-16%	-12%	dsRNA-M-77	-28%	80%
M-22-HRP	73%	19%	73%	dsRNA-M-22	-10%	49%
M-26-HRP	69%	33%	59%	dsRNA-M-26	-19%	61%
M-80-HRP	50%	48%	48%	dsRNA-M-80	-24%	38%
M-24-HRP	45%	-8%	38%	dsRNA-M-24	-29%	73%
M-9-HRP	40%	27%	55%	dsRNA-M-9	-33%	62%

M-60-HRP	38%	3%	30%	dsRNA-M-60	-15%	49%
M-83-HRP	34%	4%	-14%	dsRNA-M-83	-11%	38%
M-36-HRP	31%	9%	20%	dsRNA-M-36	-7%	22%

Table S2. Validation of dsRNA assay accuracy and precision

	Range of intervals%
Intra-batch accuracy (upper and lower limits* quality control)	-8.2%~13.6%
Intra-batch accuracy (high, medium and low quality control)	-18.3%~13.0%
Inter-batch accuracy (upper and lower limits quality control)	1.5%~3.5%
Inter-batch accuracy (high, medium and low quality control)	-1.9%~0.5%
Intra-batch precision (upper and lower limits quality control)	1.9%~14.6%
Intra-batch precision (high, medium and low quality control)	2.0%~12.5%
Inter-batch precision (upper and lower limits quality control)	6.2%~11.5%
Inter-batch precision (high, medium and low quality control)	7.4%~13.2%
Total method error (upper and lower limits quality control)	9.6%~13.0%
Total method error (high, medium and low quality control)	7.9%~13.6%

* Lower limit represents 0.046 ng/mL and upper limit represents 1.5 ng/mL.

Table S3. Validation of dilution parallelism of dsRNA assays

No.	Dilution ratio	Concentration (ng/mL)	Precision (CV)
1	20	22.2715	10.20%
	40	23.1468	
	80	25.771	
	160	26.6609	
	320	29.2268	
	640	29.0481	
2	1280	1293.6589	4.96%
	2560	1414.5823	
	5120	1503.3876	
	10240	1424.5837	
	20480	1462.4735	
3	1280	983.3696	2.50%
	2560	951.4988	

	5120	930.4627	
	10240	929.036	
	20480	916.0464	
